# Cadmium exposure induces cardiac glucometabolic dysregulation and lipid accumulation independent of pyruvate dehydrogenase activity

**DOI:** 10.1080/07853890.2021.1947519

**Published:** 2021-07-14

**Authors:** Olufemi I. Oluranti, Ebunoluwa A. Agboola, Nteimam E. Fubara, Mercy O. Ajayi, Olugbenga S. Michael

**Affiliations:** a Applied and Environmental Research Unit, Department of Physiology, College of Health Sciences, Bowen University, Iwo, Nigeria; b Cardiometabolic Research Unit, Department of Physiology, College of Health Sciences, Bowen University, Iwo, Nigeria

**Keywords:** Cadmium, cardiac, glucose, lipotoxicity, metabolism

## Abstract

**Context:**

Suppressed glucose metabolism, elevated fatty acid metabolism and lipid deposition within myocardial cells are the key pathological features of diabetic cardiomyopathy. Studies have associated cadmium exposure with metabolic disturbances.

**Objective:**

To examine the effects of cadmium exposure on cardiac glucose homeostasis and lipid accumulation in male Wistar rats.

**Methods:**

Male Wistar rats were treated for 21** **days as (*n*** **=** **5): Control, cadmium chloride Cd5 (5** **mg/kg, *p.o.*), cadmium chloride Cd30 (30** **mg/kg, *p.o*).

**Results:**

The fasting serum insulin level in this study decreased significantly. Pyruvate and hexokinase activity reduced significantly in the Cd5 group while no significant change in lactate and glycogen levels. The activity of pyruvate dehydrogenase enzyme significantly increased with an increasing dosage of cadmium. The free fatty acid, total cholesterol and triglyceride levels in the heart increased significantly with increasing dosage of cadmium when compared with the control. Lipoprotein lipase activity in the heart showed no difference in the Cd5 group but a reduction in the activity in the Cd30 group was observed.

**Conclusion:**

This study indicates that cadmium exposure interferes with cardiac substrate handling resulting in impaired glucometabolic regulation and lipid accumulation which could reduce cardiac efficiency.

## Introduction

For continuous contraction to propel blood throughout the body, the heart needs a constant and significant energy supply and, thus, has established an elaborate metabolic system for the use of all carbon substrates. It relies mainly on the fatty acid oxidation (∼60–90%) and to a lesser degree on glucose (∼10–40%), ketone bodies, lactate for its energy output [1]. However, the myocardial metabolic mechanism is extremely versatile, allowing a range of physiological and pathological conditions to alter the choice of the substrate. While it is thought that a cardiac metabolic transition is an adaptive response to the availability of cardiac substrate. Evidence supports the belief that the preference for myocardial substrate changes may play a causal role in cardiac dysfunction [2,3]. The heart's energy supply is met by the combined use of various substrates. Nevertheless, glucose and fatty acids constitute the main energy sources under aerobic conditions, with fatty acids contributing a greater part of this energy supply [4–6]. Lipoproteins are the primary sources of cardiac tissue fatty acids, and lipoprotein lipase (LPL) is the rate-limiting triglyceride hydrolysis found in these molecules [7]. Disorders in the metabolism and energy of cardiac substrates have been reported to be the major contributors to cardiac dysfunction like diabetic cardiomyopathy (DCM) [8,9].

Lipotoxicity is characterized as the accumulation in tissues and organs of lipid intermediates and final products in which it normally does not accumulate. It is thought that lipotoxicity plays a major role in heart disease, obesity and diabetes [10]. Excess lipid accumulation in the heart called cardiac lipotoxicity can cause alterations in the cell as lipids are crucial regulators of the heart function through their role in the structure of the membrane, transport, signalling and β-oxidation substrate for mitochondrial energy production [11,12]. Together with excessive accumulation of intra-myocellular triglycerides (TG), cardiac lipotoxicity often includes changes in various lipid groups and their fatty acid composition, thus, aiding the development of active lipid mediators that influence metabolism and cardiac function, partly by distorting mitochondrial function [13–15].

Studies have documented stimulating evidence connecting environmental exposure to heavy metals with increased risks of cardiovascular disease, hypertension, metabolic syndrome and diabetes [16,17]. Cadmium (Cd), a heavy metal and an environmental pollutant, known to cause toxic effects in humans at relatively low exposure in all its biological types [18]. This pollutant, however, is ubiquitous, not biodegradable and has a long environmental life [19]. Cd accumulates in the human body, particularly in the liver and kidneys, which are vital organs for acute toxicity-induced detoxification processes, causing severe health problems [20,21]. The Joint FAO/WHO Expert Committee on Food Additives (2004) set a provisional tolerable intake of Cd at 7** **μg kg^−1^ body weight per week. In this sense, as a result of growing levels of toxic metals and contaminants in terrestrial and marine environments from natural and anthropogenic sources, the importance of monitoring the impact of toxic elements on living organisms has increased in recent years. Water, soil, environment, plants, livestock, vegetables, meat and seafood are all showed by survey data to be the possible risk of exposure to Cd [22]. Cd-induced toxicity is mediated by free radical generation and subsequent accumulation of reactive oxygen species (ROS), which plays a crucial role in tissue damage [23]. The major events caused by cadmium poisoning include oxidative stress, cell cycle progression, DNA damage and apoptosis [24]. A correlation has been identified between exposure to Cd and the possibility of cardiovascular diseases such as hypertension, myocardial infarction, cardiomyopathy, stroke, heart failure, arteriosclerosis and peripheral arterial disease [25].

Diabetic cardiomyopathy (DCM) is mostly due to diabetes and is independent of hypertension and coronary artery disease, leading to initial diastolic and later-stage systolic dysfunction [26]. Elevated fatty acid metabolism, lipid deposition and suppressed glucose metabolism within myocardial cells are the key pathological features of DCM [27]. Studies have shown Cd affected blood insulin levels and glucose metabolism by inducing changes in islet function, insulin secretion and insulin activity [28,29]. However, studies on Cd-induced cardiac disruption of glucose homeostasis are scarce. Therefore, this study aimed at investigating the effect of Cadmium exposure on cardiac glucose metabolism and lipid accumulation in male Wistar rats.

## Materials and methods

### Experimental animals and protocol

By following the guidelines of the National Institutes of Health Guide for the Treatment and Use of Laboratory Animals, all experimental procedures were approved by the Bowen University Ethical Review Committee with the protocol number BUTH/REC-101 and every attempt was made to reduce both the number of animals used and their suffering. Fifteen (15) Wistar rats, approximately 150** **g body weights, were procured from the Central Animal House, College of Health Sciences, Bowen University, Iwo, Nigeria. The animals were fed with standard rat feed and water *ad libitum* under a condition of 12-hour light/dark cycle daily. The rats were acclimatized for 7 days before they were subjected to different treatments. The animals were weighed every week and the doses were adjusted accordingly. All 15 animals were randomized into 3 groups of 5 rats each and were treated as below for 21** **days:

- Group 1: Control
- Group 2: Cadmium chloride Cd5 (5mg/kg, *p.o*)
- Group 3: Cadmium chloride Cd30 (30mg/kg, *p.o*)

About 4 ml of blood was collected *via* cardiac puncture after the post-exposure days into a plain tube under mild ethyl ether anaesthesia. By cervical dislocation, animals were sacrificed and opened by abdomen-pelvic incision. The heart, free of adipose tissue, blotted dry and measured, was carefully removed. The hearts were homogenized at 4 times phosphate buffer weight/volume (pH 7.4), centrifuged at 10,000** **rpm at 40 °C for 15** **min and supernatant fraction for biochemical assay.

### Biochemical analysis

#### Plasma glucose and insulin

The amount of plasma glucose was estimated using a commercial kit (Sigma Diagnostics Pvt. Ltd., Baroda, India) and using the Trinder process [30]. An ELISA kit (Boeheringer-Manneheim Kit, Mannheim, Germany) was used for plasma insulin.

#### Tissue glycogen

About 100** **mg of heart tissue was made to undergo alkali digestion for this assay (5** **ml of 30 percent w/v KOH) for 20** **min in a boiling water tank and cooled down to room temperature. 3.0** **ml of absolute ethanol was applied, followed by a drop in NH_4_CH_3_CO_2_. In order to collect the precipitate, the resulting mixture was centrifuged at 3000*g* for 10** **min. The precipitated glycogen sample aliquot reacted with 4** **ml of anthrone reagent heated for 20** **min in a boiling water bath. The content of cardiac glycogen (green colour) was measured at 640** **nm and expressed as wet tissue mg/g.

#### Tissue lactate and pyruvate

Lactate and pyruvate tissue levels were measured by a standardized colorimetric procedure using Fortress Diagnostics Ltd. assay kits (Antrim, UK).

#### Hexokinase

Hexokinase activity, an enzyme for glucose metabolism, was assayed according to [31]. The total reaction mixture consists of 5.3** **ml of glucose (1** **ml, 5** **mM), adenosine triphosphate (ATP) (0.5** **ml, 4** **mM), KCI (0.4** **ml, 7** **mM), potassium dihydrogen phosphate (0.4** **ml), magnesium chloride (0.1** **ml, 100** **mM), sodium fluoride (0.4** **ml), and 50 mM Tris-HCl buffer (2.5** **ml, pH 8.0). At 37 °C for 5** **min, the reaction mixture was pre-incubated, followed by the addition of heart homogenate (2** **ml) to initiate the reaction. A volume of 1** **ml was immediately transferred to the TCA-containing tubes (10 percent w/v, 1** **ml) and then incubated at 37** **°C for 30** **min. To estimate hexokinase activity, the remaining glucose content in the supernatant was calculated at 340** **nm following centrifugation.

#### Pyruvate dehydrogenase

The activities of PDH were measured spectrophotometrically according to the method of [32].

#### Cardiac triglyceride, cholesterol, phospholipid, free fatty acid and lipoprotein lipase

These were all measured and estimated with a commercial assay kit using enzymatic colorimetric test.

#### Statistical analysis

The results are expressed as a mean ± standard error of the mean (SEM). Using Graph Pad Prism 5.03 (GraphPad Software, La Jolla, CA), statistical analysis was carried out. Using a one-way ANOVA and post hoc test (Tukey's test), the findings was analyzed, considering *p*** **≤** **0.05 as statistically significant.

## Results

 

## Discussion

The optimum functioning of the heart is closely linked to its metabolic capacity and cardiac lipotoxicity is a well-established feature in the context of obesity and diabetes which is associated with cardiac damage. Our study investigated the effect of an environmental pollutant, cadmium, on cardiac glucose oxidation, metabolites and lipid status.

Cadmium (Cd) increased the relative organ weight as noted in this study which is similar to the work of Ivanova and co-workers where the administration of cadmium to mice over 2** **weeks lead to a significant increase in the heart weight by 20% compared to the controls [33]. Fluoride administration was also reported to increased organ weight and this may be associated with lipid accumulation and inflammation process triggered by this metal [34,35].

The fasting serum insulin level in this study decreased significantly in Cd5 and Cd30 groups compared with the control which is in line with previous studies [36–38]. One of the main sites of Cd accumulation is the pancreas [39]. The drop in fasting insulin levels in Cd-exposed animal could therefore indicates a potential direct pancreatic toxic effect of cadmium. Histological analysis showed a significant decrease in Cd-exposed mice in the islets relative area [38]. Zinc (Zn^2+^) plays a key role in the process, storage, release and action of insulin in beta-cells in the pancreas. Insulin is collected in vesicles, where two Zn^2+^ ions co-ordinate with six insulin monomers to create a hexameric structure and insulin crystals are based on it [40]. Cadmium vies for multiple binding sites with Zn^2+^ ions. Cadmium as well uses zinc transporters present in various cells, including β-cells, for the purpose of transport through the cells, where there is higher concentration of Zn^2+^. Kinetic experiments have shown that heavy metal transporters ZIP8 and ZnT are commonly used by Zn^2+^ ions for transport purposes [41]. These carriers have a high cadmium affinity for [28]. In addition, ATP gotten from the process of glycolysis causes the closure of potassium channels responsive to ATP, ensuing in cellular depolarization with concomitant opening of voltage-dependent calcium channels. The flux of calcium from these opened channels causes insulin to be released from the β-cells [42]. Cadmium can block calcium channels and then prevent the release of insulin from the pancreas [43].

Unexpectedly in this study, there was a significantly reduction (*p*** **<** **0.01) in the glucose level in both cadmium treated groups compared with the control despite the reduction in insulin level. This is in accordance with Li and colleague’s finding where decreased insulin secretion and unaffected glucose homeostasis in male C57BL/16 mice exposed to cadmium was observed [38]. The rationale for the difference between reduced insulin secretion and decreased glucose level is unclear. It is likely that the changes in the islet in cadmium-dosed rat were not much enough to have a physiological effect on glucose homeostasis. It could also be that despite the reduction in insulin level, the available insulin was able to regulate the blood glucose in liver uptake and utilization. No observable changes in the cardiac level of glycogen and lactate but a significant reduction was observed in the Cd5 group when compared with the control while there was no change in the pyruvate level in the Cd30 group.

Hexokinase activity in the heart reduced significantly (*p*** **<** **0.05) in the Cd5 group when compared with the control while the activity was not statistically different in the Cd30 group as against the control. This result support earlier report where the activity of hexokinase in the heart and liver reduced following Cd-treatment [44]. Hexokinase is the first glycolysis enzyme that phosphorylates glucose, the first and rate-limiting stage of the glycolytic pathway, by moving the phosphoryl group from ATP to glucose-6-phosphate. It is dependent and sensitive to insulin and is almost completely inhibited or inactivated in the diabetic rat liver in the absence of insulin [45].

Streptozotocin-cadmium-induced diabetic rats caused β-cell necrosis and degeneration, leading to insulin secretion deficiency and hexokinase activity dysfunction due to reduced glycolysis and glucose usage for energy production [46,47]. The effect of cadmium on glycolysis in the upper part of the skeletal muscles in mice was investigated and it was found that phosphofructokinase and hexokinase were inhibited by Cd [48]. Due to their high attraction for free electron pairs in cysteine -SH groups, all enzymes are inhibited by heavy metals *via* the same way, and this is critical in enzyme function. A large number of candidate cysteine residues were present in the structural study of hexokinase and phosphofructokinase [49]. In addition, some metals like cadmium can alter the structure of macromolecules [50]. Cadmium brings conformational changes with corresponding increased *K*_m_ value to the structure of glucokinase in the liver; this means that cadmium reduces the affinity between glucose and glucokinase [51].

In diabetic cardiomyopathy (DCM), as shown by animal models, cardiac glycolysis and pyruvate oxidation are both affected. The mechanisms involved in reducing the oxidation of glucose in the DCM also include decreased expression and activity of essential enzymes of glucose oxidation, including G6P and PDH [52]. There is an accumulation of glycolytic intermediates as a result of decreased myocardial glycolysis and glucose oxidation in DCM. These glycolytic intermediates are linked to increased production of reactive oxygen species (ROS) [53], decreased expression of calcium ATPase 2a (SERCA2a) in the sarcoplasmic reticulum [54] and increased accumulation of Ca2+, all contributing to cardiac dysfunction. However, the increase in the activity of the pyruvate dehydrogenase (PDH) enzyme in the heart is unexplained in this report, which needs further investigation. Elevated levels of circulating free fatty acids in obesity and diabetes increase the availability of fatty acids in the heart, where increased oxidation of fatty acids will minimize glucose oxidation by impairing the activity of pyruvate dehydrogenase (PDH), leading to a decrease in energy efficiency [55].

The free fatty acid (FFA), triglyceride (TG) and total cholesterol levels in the heart increased significantly with increasing dosage of cadmium when compared with the control. This finding corroborates previous reports where serum and cardiac TG increased in diabetic rats [56]. A significant increase in cardiac TG levels of normotensive obese animals have been observed in experimental and clinical studies [57–60]. In correlation with earlier observation, an increase in FFAs was also observed in obese rats [61]. Histology and cardiac tissue findings have also shown that within cardiomyocytes, DCM hearts mount up lipid drops, followed by elevated levels of FFA [62]. Induced by a mismatch between fatty acid uptake and oxidation, the accumulation of triglycerides in the heart develops as a determinant of cardiac damage, primarily through accumulation of free radicals and successive activation of apoptosis [63]. Free fatty acids serve as effective fuel molecules for myocardial contractions, but higher FFA level may also take part in CVD pathology [64]. FFAs may disrupt ion channels and membrane integrity due to amphiphilic and detergent-like properties [65], uncouple mitochondrial enzymes, thus, reducing the efficiency of the respiratory cycle and undermining the heart's contractile function [66].

Cardiac lipotoxicity, a well-established characteristic in obesity or diabetes, is the accumulation of lipid droplets within cardiomyocytes [63]. Increased myocardial FFA uptake may be due to cardiac lipotoxicity. Cardiomyocytes can take up FFA in two main ways: 80% of FFA is absorbed through protein-mediated transport while 20% of FFA is by passive diffusion [67]. Long chain fatty acids (LCFA) are absorbed into cardiomyocytes by fatty acid translocase (CD36) and internal membrane LCFA can stimulate peroxisome proliferator activated receptor (PPARa), which can lead to transcriptional upregulation of the enzymes involved in the transport of FA (FAT/CD36) and FA oxidation (CPT-1), which in turn exacerbates cardiac lipid disorder [5]. Adenosine monophosphate kinase (AMPK), a heterotrimeric enzyme triggered within cardiomyocytes by an increase in the AMP/ATP ratio, is a regulator involved in the metabolism of glucose and lipids [68]. By regulating CPT-1 *via* phosphorylation of acetyl CoA carboxylase and liberating of malonyl-CoA-mediated CPT-1 inhibition, AMPK can modulate cardiac FFA metabolism [69].

Lipoprotein (LPL) is a lipoprotein and energy metabolism rate limiting enzyme that catalyzes the hydrolysis in the blood of TG rich lipoproteins such as VLDL and chylomicrons [70]. As noted in this study, there has also been a decrease in LPL activity in both animal and human diabetes due to insulin deficiency, because its synthesis is caused by insulin [71]. A similar effect was reported when doxorubicin induced cardiotoxicity by inhibiting cardiac fatty acid oxidation with a decrease in cardiac LPL activity in the heart [72]. The decreased LPL activity in the heart could be due to low lipoprotein degradation [73]. A defective LPL secretion has been suggested to contribute to decreased lipolytic activity, leading to increased triglycerides and phospholipids. The association of tissue hypertriglyceridaemia with cardiovascular disturbances observed in cadmium-exposed rats was associated with decreased LPL function, a key enzyme in TG hydrolysis [74].

## Conclusion

This study shows that cadmium exposure interferes with cardiac substrate handling resulting impaired glucometabolic regulation and lipid accumulation associated with elevated pyruvate dehydrogenase and reduced lipoprotein lipase activity. Therefore, cadmium induces cardiac damage through substrate mishandling and lipotoxicity.

**Figure 1.**
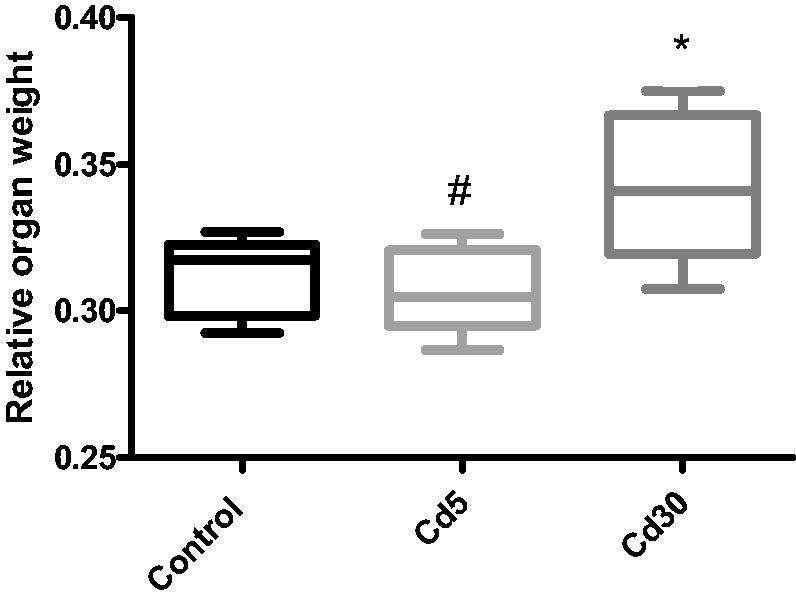
Effect of cadmium on relative organ (heart) weight. The relative organ weight increased significantly (*p < 0.05) in the Cd30 group as compared to the control while it decreases significantly (^#^*p* < 0.05) in the Cd5 group when compared with the Cd30 group.

**Figure 2.**
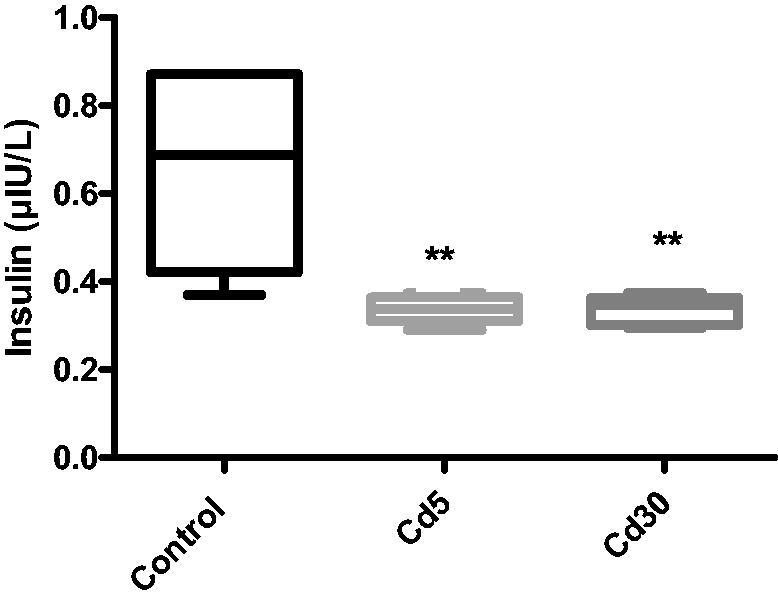
Effect of cadmium on serum insulin level. The insulin decreased significantly (**p < 0.01) in Cd5 and Cd30 groups compared with the control.

**Figure 3.**
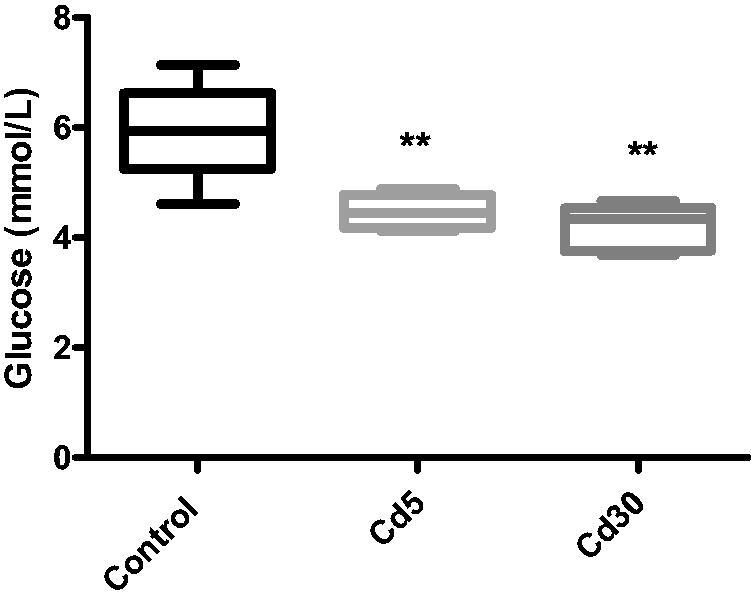
Effect of cadmium on serum glucose level. There is a significantly reduction (**p < 0.01) in the glucose level in both cadmium treated groups compared with the control.

**Figure 4.**
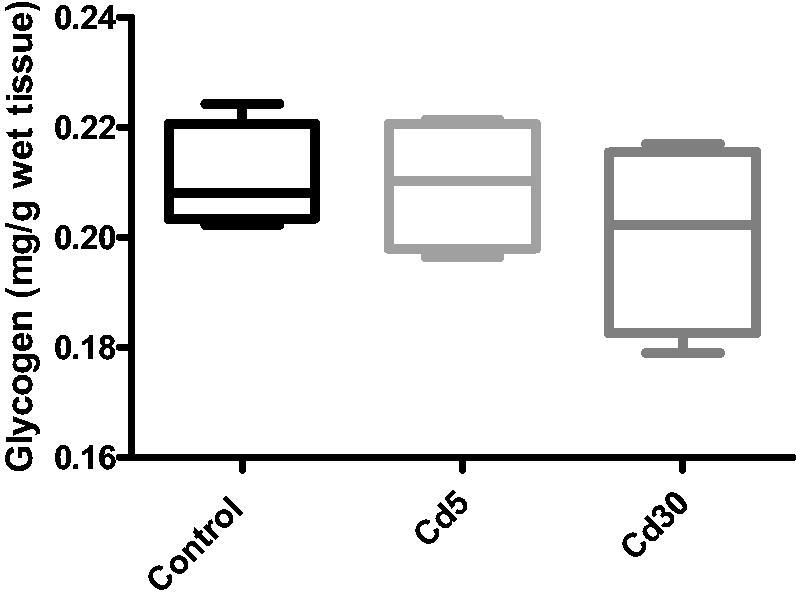
Effect of cadmium on glycogen content in the heart. No observed significant changes in the glycogen content in the heart between the control and the treated groups.

**Figure 5.**
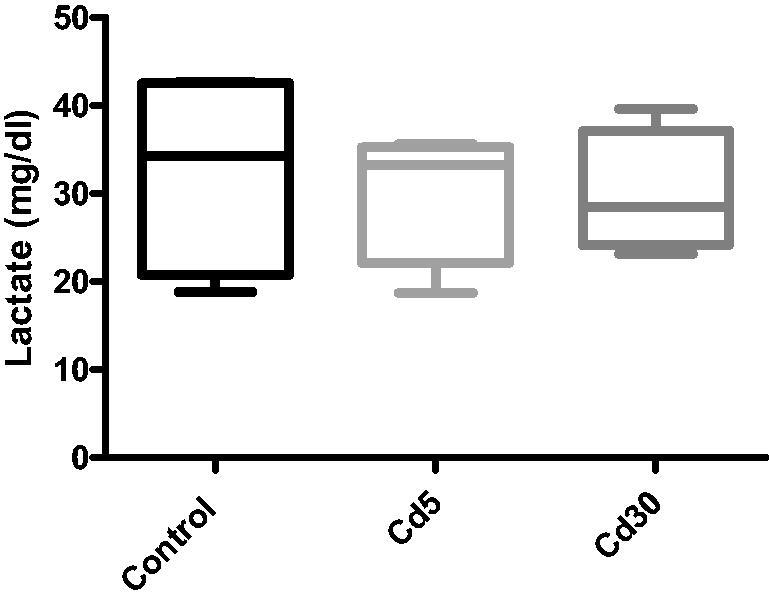
Effect of cadmium of lactate level in the heart. There were no observable statistical changes in the lactate level in the heart between the control and the treated groups.

**Figure 6.**
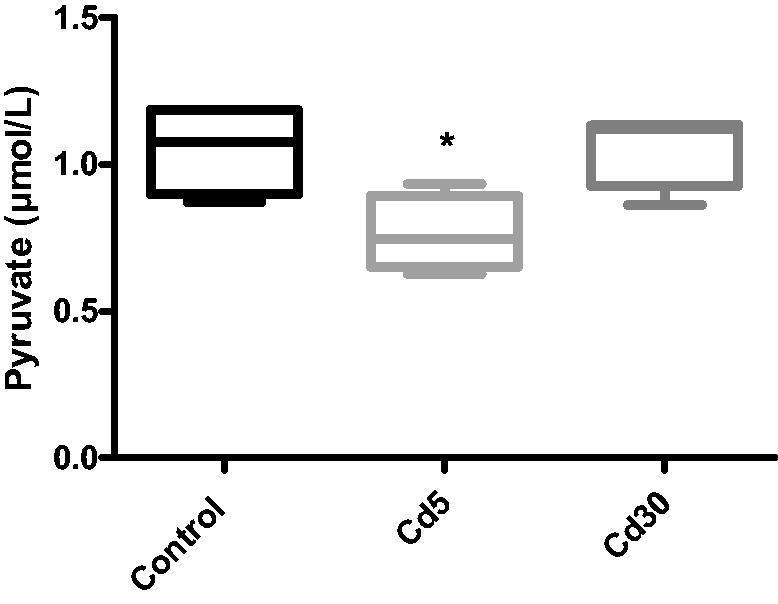
Effect of cadmium on cardiac pyruvate level. A significant reduction was observed in the Cd5 group when compared with the control while there was no change in the pyruvate level in the Cd30 group.

**Figure 7.**
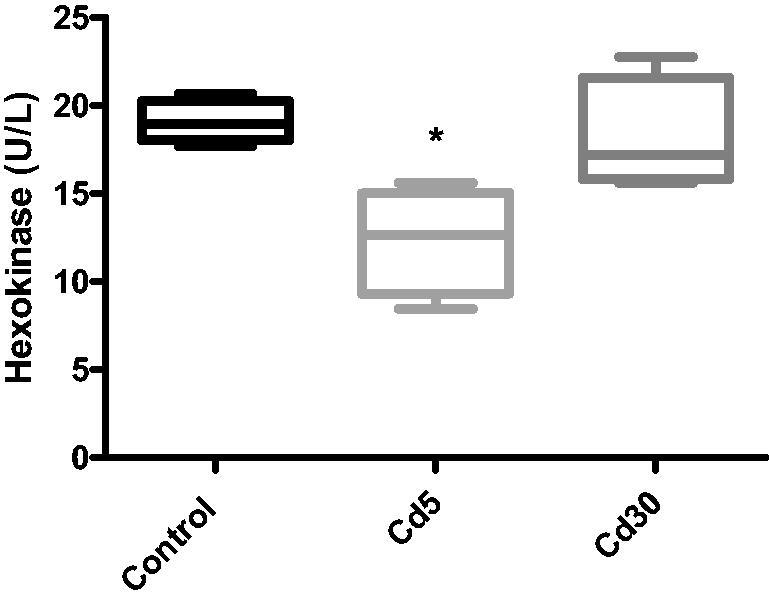
Effect of cadmium on hexokinase activity in the heart. Hexokinase activity reduced significantly (*p < 0.05) in the Cd5 group when compared with the control while the activity was not statistically different in the Cd30 group as against the control.

**Figure 8.**
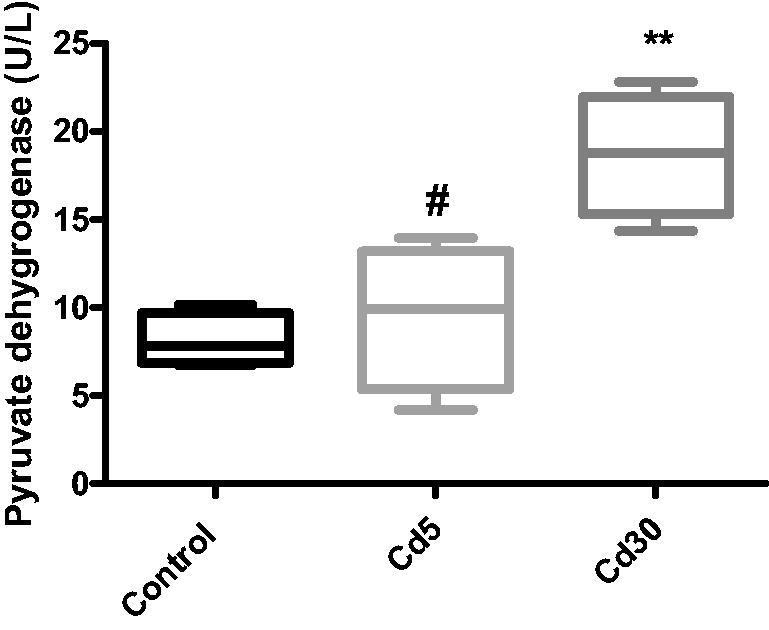
Effect of cadmium on cardiac pyruvate dehydrogenase activity. The activity of pyruvate dehydrogenase enzyme significantly increased with increasing dosage of cadmium (*p < 0.05 compared with the control, #*p* < 0.05 compared with the Cd30 group).

**Figure 9.**
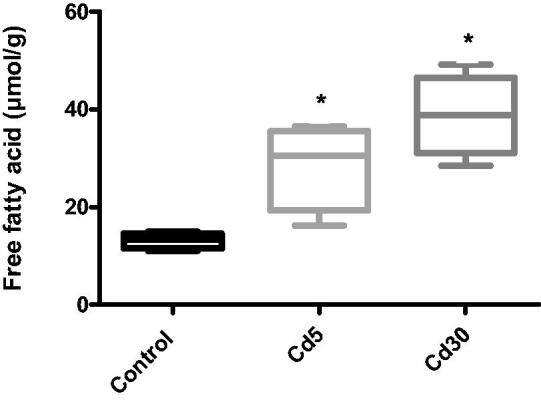
Effect of cadmium on cardiac free fatty acid level. The free fatty acid level in the heart increased significantly with an increasing dosage of cadmium when compared with the control (*p < 0.05).

**Figure 10.**
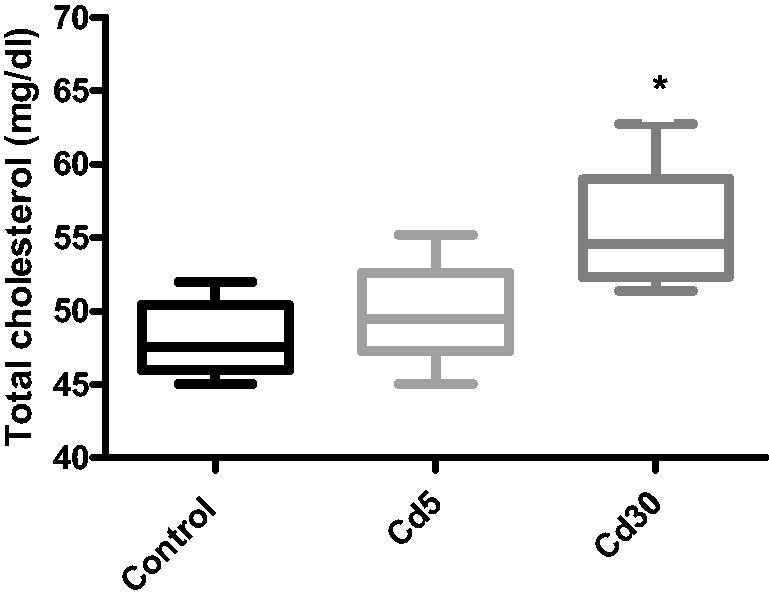
Effect of cadmium on cardiac total cholesterol level. There was an increase in the total cholesterol level in the heart as the dosage of cadmium increase. However, the increase was statistically significant (*p < 0.05) in the Cd30 group when compared with control.

**Figure 11.**
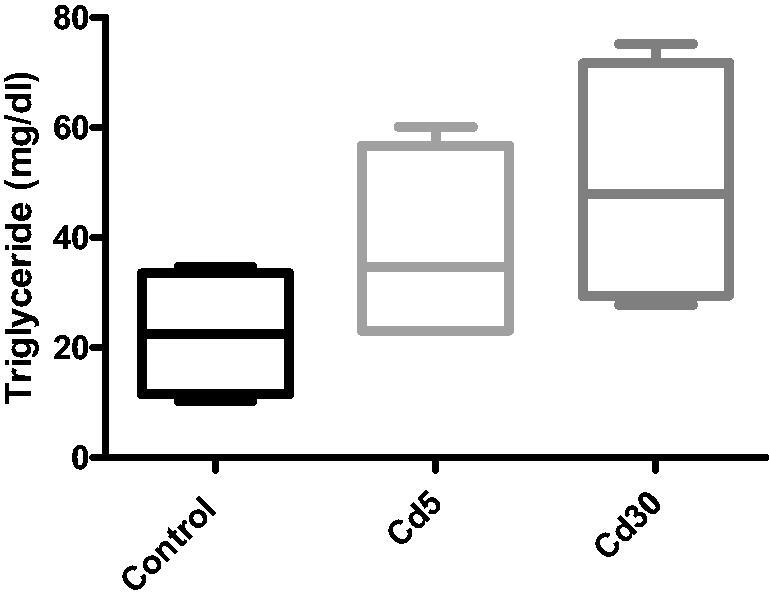
Effect of cadmium on cardiac triglyceride level. The triglyceride level increased with increasing dosage of cadmium, although not statistically significant.

**Figure 12.**
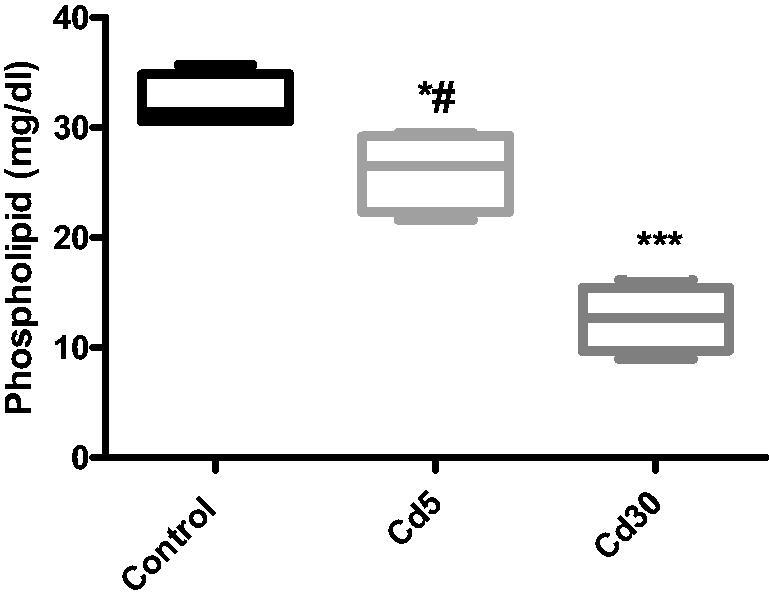
Effect of cadmium on cardiac phospholipid level. The phospholipid level in the heart of rat treated with cadmium decreased significantly with increasing dosage of cadmium (*p < 0.05, ***p < 0.001 when compared with the control; ^#^*p* < 0.05 when compared with the Cd30 group).

**Figure 13.**
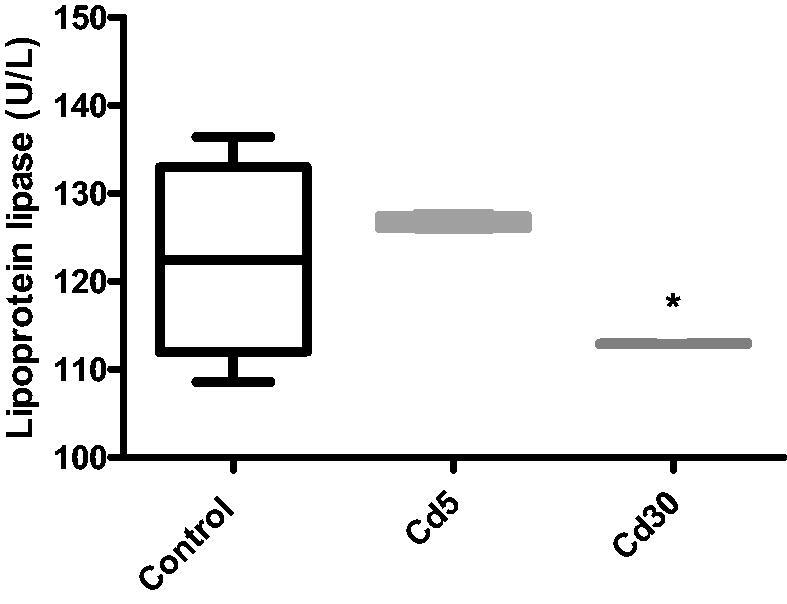
Effect of cadmium on cardiac lipoprotein lipase activity. Lipoprotein lipase activity in the heart showed no difference in the Cd5 group compared with control but a statistical (*p < 0.05) reduction in the activity in the Cd30 group was observed compared with control.

**Figure 14.**
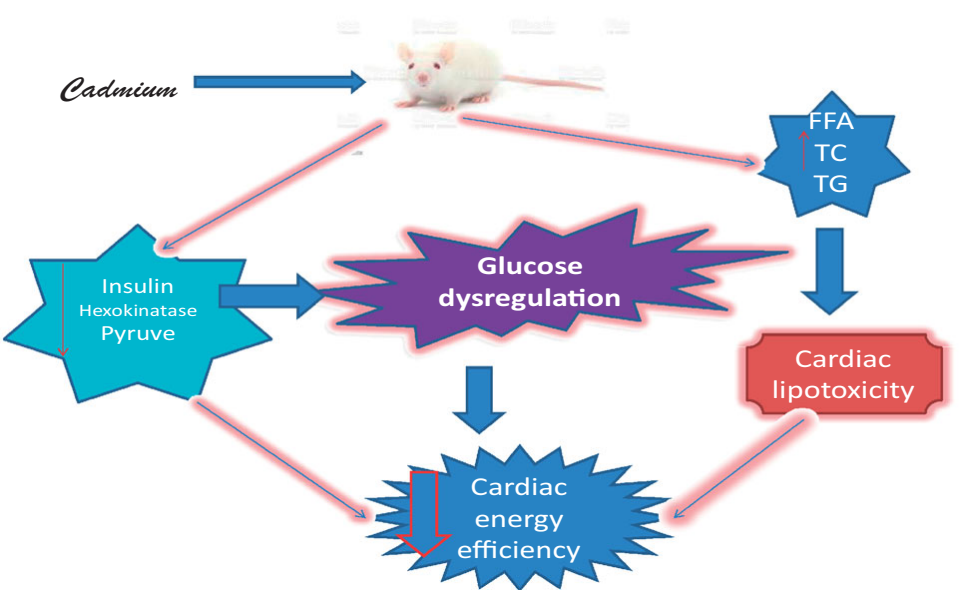
Schematic diagram illustrating the likely pathway through which cadmium induces glucometabolic dysregulation. FFA: free fatty acid; TG: triglyceride; TC: total cholesterol.

## Data Availability

The data that support the findings of this study are available from the corresponding author [O.I.O] upon reasonable request.
